# Preventive effect of fluvastatin on the development of medication-related osteonecrosis of the jaw

**DOI:** 10.1038/s41598-020-61724-6

**Published:** 2020-03-27

**Authors:** Naomi Adachi, Yasunori Ayukawa, Noriyuki Yasunami, Akihiro Furuhashi, Mikio Imai, Koma Sanda, Ikiru Atsuta, Kiyoshi Koyano

**Affiliations:** 1 Section of Implant and Rehabilitative Dentistry, Division of Oral Rehabilitation, Faculty of Dental Science, Kyushu University, Fukuoka, Japan; 2 Division of Advanced Dental Devices and Therapeutics, Faculty of Dental Science, Kyushu University, Fukuoka, Japan; 3 Present Address: Department of Reconstructive Science, School of Dental Medicine, University of Connecticut, CT, USA

**Keywords:** Dental diseases, Oral surgery, Bisphosphonates in dentistry

## Abstract

Medication-related osteonecrosis of the jaw (MRONJ) occurs in patients undergoing oral surgery while medicated with bisphosphonate, denosumab or anti-angiogenic agents. We employed a MRONJ-like rat model to investigate whether injecting fluvastatin at extraction sites prevents MRONJ-like lesion. A MRONJ-like model was created by treating rats with zoledronate and dexamethasone, extracting teeth, and immediately injecting fluvastatin at the extraction site. The experimental group comprised three subgroups treated with low (0.1 mg/kg; FS-L), medium (1.0 mg/kg; FS-M) and high concentrations (10 mg/kg; FS-H) of fluvastatin. Necrotic bone exposure was significantly lower in the FS-M (*p* = 0.028) and FS-H (*p* = 0.041) groups than in the MRONJ group. The distance between the edges of the epithelial surfaces was significantly shorter in the FS-M (*p* = 0.042) and FS-H (*p* = 0.041) groups. The area of necrotic bone and the necrotic bone ratio were significantly smaller in the FS-H group (*p* = 0.041 and *p* = 0.042 respectively). Bone volume fraction calculated on μ-CT images was significantly larger in the FS-H group than in the MRONJ group (*p* = 0.021). Our findings suggest that a single local injection of fluvastatin following tooth extraction can potentially reduce the chance of developing MRONJ-like lesion in rats.

## Introduction

Bisphosphonates are drugs that inhibit bone resorption by suppressing osteoclast activity. Bisphosphonates have been prescribed for osteoporosis, prevention of bone metastases from some types of carcinoma and multiple myeloma, Paget’s disease, and some other conditions^1^. Bisphosphonates have been prescribed for these conditions for more than two decades with relatively few recognised adverse drug reactions; however, exposure of the jaw bone associated with administration of bisphosphonates was reported in 2003^2^. Since then, many similar reports have been published^3–5^. Additionally, anti-receptor activator of nuclear factor κB ligand (denosumab) has also been reported to induce bone exposure^6,7^. This condition has been named medication-related osteonecrosis of the jaw (MRONJ)^8^.

The prevalence of MRONJ is reported to be 1–15% in patients with carcinoma receiving high dose bisphosphonates or denosumab and 0.001–0.01% in patients with osteoporosis receiving low dose bisphosphonates^9^. Given that bisphosphonates are extensively used worldwide, tens of millions of patients are exposed to the risk of developing MRONJ.

Proposed treatments for MRONJ have included irrigation^10^, administration of antimicrobial agents^11,12^, and debridement^10,11^; however, these treatments only address the symptoms. It was recently reported that parathyroid hormone alleviates the symptoms of MRONJ^13,14^; however, hormone treatment is problematic and this treatment has not been widely implemented. Other proposed treatment modalities include laser therapy^15,16^, hyperbaric oxygen therapy^17^, and platelet-rich plasma therapy^16,18^; however, no effective treatment protocol has yet been established.

The mechanism underlying development of MRONJ remains to be clarified; it is believed that infection and subsequent inflammation play crucial roles^19,20^. Other proposed mechanisms have included suppression of bone remodelling^21^, angiogenesis^22–24^, proliferation of oral mucosal cells^25,26^, and disordered immune function^27,28^.

Statins, 3-hydroxy-3-methyl-glutaryl-coenzyme A (HMG-CoA) reductase inhibitors, are widely prescribed for patients with hyperlipidaemia. Other reported functions of statins include stimulation of bone formation^29,30^, anti-inflammatory effects^31,32^, antimicrobial activity^33,34^, and angiogenesis^35,36^. In our previous study, we found that statins promote healing of tooth extraction sockets by healing both gingival soft tissue and alveolar bone^37^.

In the present study, we expected that statins would reduce the risk of the development of MRONJ-like lesion by promoting bone and gingival healing via their antimicrobial function and promotion of angiogenesis. The null hypothesis of the present study was that fluvastatin has no preventive effect on the development of MRONJ-like lesion. To investigate this hypothesis, we established a MRONJ-like rat model in which we administered a single injection of fluvastatin in the vicinity of tooth extraction sockets to determine whether statins can prevent MRONJ-like lesion.

## Methods

### Animals

Thirty female Wistar rats (4 weeks old, weight 70–90 g, five groups of six) were used in all experiments. A required sample size of six was determined by performing power analysis with an effect size of 0.8, statistical significance 0.05, and power 0.8. The effect size was determined based on a previous study^37^.

All experiments were performed in accordance with the ARRIVE Guidelines for reporting animal research^38^. All procedures involving experimental animals were approved by the Institutional Animal Care and Use Committee of Kyushu University (Approval Number: A30-369-0) and complied with the Guide for the Care and Use of Laboratory Animals (7th and 8th edition, ILAR-NRC).

### MRONJ-like model

A MRONJ-like rat model was established based on a previous report by Kaibuchi *et al*.^39^. In brief, percutaneous injections of 66 μg/kg of zoledronate (BP; Zometa; Novartis Pharma, Tokyo, Japan) and 5 mg/kg of dexamethasone (Dex; Decadron; Aspen Japan, Tokyo, Japan) were administered three times a week until the end of the experiment (n = 6). Control animals were percutaneously injected with the same volume of saline (n = 6). MRONJ-like model and control animals were randomly assigned. Two weeks after starting BP and Dex (or saline) administration, the right maxillary first molar was extracted under general anaesthesia with intraperitoneal injection using 0.15 mg/kg of medetomidine hydrochloride (Medetomin; Meiji Seika Pharma, Tokyo, Japan), 2 mg/kg of midazolam (Dormicum; Maruishi Pharmaceutical, Osaka, Japan) and 2.5 mg/kg of butorphanol tartrate (Vetorphale; Meiji Seika Pharma). Two weeks after the extraction, the animals were killed with 12 mg/100 g of pentobarbital sodium (Somnopentyl; Kyoritsu Seiyaku, Tokyo, Japan).

### Application of fluvastatin

Fluvastatin (fluvastatin Sodium n-Hydrate; Fujifilm Wako Pure Chemical, Osaka, Japan) was dissolved in 0.1 ml saline per rat. Three concentrations of fluvastatin were tested, namely, low concentration (0.1 mg/kg; FS-L group, n = 6), medium concentration (1.0 mg/kg; FS-M group, n = 6) and high concentration (10 mg/kg; FS-H group, n = 6). The fluvastatin was injected from the buccogingival fold into the vicinity of the extraction socket immediately after tooth extraction in the MRONJ-like rats that had been receiving BP and Dex as described above. Two weeks after the tooth extraction and injection of fluvastatin, all animals were killed as described above. All fluvastatin groups were compared with a MRONJ group that did not receive fluvastatin (n = 6).

### μ-CT examination and morphometry

After the rats had been killed, their maxillae were dissected out and fixed in 10% paraformaldehyde (Merck, Darmstadt, Germany) for 24 h, then examined by μ-CT (SkyScan 1076; Bruker microCT, Kontich, Belgium; tube current: 201 μA; voltage: 49 kV; pixel size: 9 μm). Bone volume fraction (bone volume/tissue volume: BV/TV) at the extraction socket was measured^40^ using image analysis software (CTAn; Bruker microCT, Kontich, Belgium).

### Histology and histomorphometry

After examination by μ-CT, the maxillae were demineralised with 20% ethylenediaminetetraacetic acid (Dojindo Laboratories, Kumamoto, Japan) and dehydrated with a graded ethanol series (99% Synthetic Ethanol; Mitsubishi Chemical, Tokyo, Japan) and xylene (Nacalai Tesque, Kyoto, Japan), then embedded into paraffin. Sections (3 μm thick) were cut parallel to the coronal plane and stained with Ladewig’s fibrin stain^37,41^. Sections were examined by light microscopy (BZ-9000; Keyence, Osaka, Japan). The length of necrotic bone exposed toward the oral cavity, distance between the edges of the epithelial surfaces, area of necrotic bone and necrotic bone ratio were calculated for three sections of the maxillae, namely the midsection of the extraction socket and sections 100 μm mesial and distal to the midsection. The area of necrotic bone was defined as the area of the bone with empty osteocytic lacunae. The necrotic bone ratio was defined as the ratio of empty to occupied osteocytic lacunae within a representative square of 2500 μm^2^ at the exposed area of the bone^42^. All measurements were done three times and the average calculated.

### Statistical analysis

An *a priori* Shapiro-Wilk test was performed to test for normality. If normality was rejected, a non-parametric test was employed. Student’s *t*-test was used to compare two sets of parametric data and the Mann-Whitney *U*-test to compare non-parametric data. When pairwise comparisons between the MRONJ group and each experimental group were performed, Williams’ test was employed for parametric data and Steel’s test for non-parametric data. All statistical analyses were performed using Microsoft Excel statistical add-in (BellCurve for Excel 2.15, Social Survey Research Information, Tokyo, Japan). Differences were considered statistically significant if *p* < 0.05 (except for Williams’ test, which was one-sided; here the significance level was set as *p* < 0.025).

## Results

### Development of the MRONJ-like model

**Figure 1:**
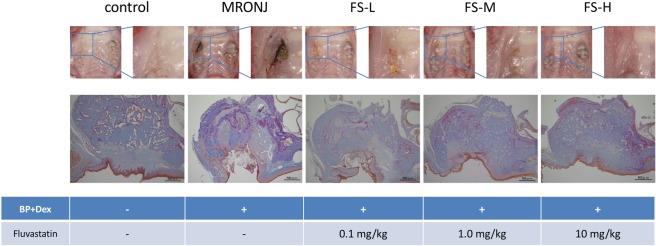
Intraoral and histological findings according to group. Two weeks after extraction of the right maxillary first molar, epithelial continuity has been restored in the control group; however, exposed necrotic bone is present in the MRONJ group. The area of newly-formed bone is smaller in the MRONJ group than in the control group. Among the groups who received fluvastatin, closure of the extraction socket by soft tissue was observed only in the FS-H group.

**Figure 2:**
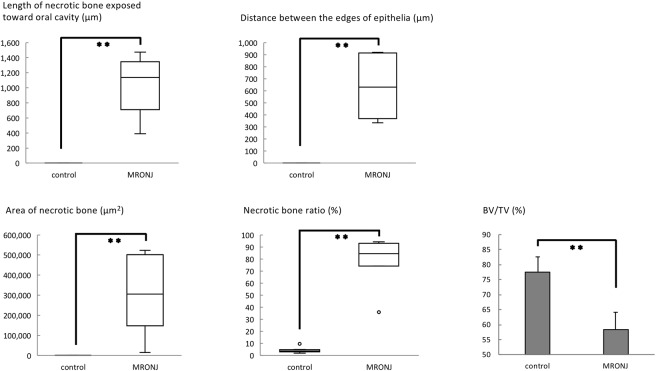
Five variables for evaluating MRONJ-like status. Two weeks after extraction of the right maxillary first molar, four variables were measured on histological sections, namely the length of necrotic bone exposed toward the oral cavity, distance between the edges of the epithelial surfaces, area of necrotic bone, and necrotic bone ratio. All four of these variables were significantly larger in the MRONJ group than in the control group. Mann–Whiney *U-*test. **p < 0.01. However, BV/TV measured on μ-CT images was significantly smaller in the MRONJ group. Student’s *t*-test. **p < 0.01.

First, the MRONJ-like rat model was set up. Two weeks after the tooth extractions, all extraction sockets in rats that had received zoledronate and dexamethasone (BP + Dex) had exposed necrotic bone with incomplete restoration of epithelial continuity, insufficient formation of connective tissue, and infiltration of white blood cells. Necrotic bone spread from the exposure site but was not detected in the periapical area. No necrotic bone was detected in the periapical and periodontal regions around the corresponding non-extracted teeth. In contrast, in the control group, both restoration of epithelial continuity and formation of osteoid were observed at the socket. The bone was completely covered with epithelium, and connective tissue formation was observed. No necrotic bone was detected around the extraction socket or bone surrounding the corresponding non-extracted teeth (Fig. 1, two rows from left). A significantly longer length of necrotic bone was exposed toward the oral cavity in the BP + Dex group than in the control group (Mann–Whitney *U*-test, *p* < 0.001) (Fig. 2). The distance between the edges of the epithelial surfaces was also significantly longer in the BP + Dex group than in the control group (Mann–Whitney *U*-test, *p* < 0.001) (Fig. 2). The area of necrotic bone was significantly larger in the BP + Dex group than in the control group (Mann–Whitney *U*-test, *p* < 0.001) (Fig. 2). The necrotic bone ratio was also significantly larger in the BP + Dex group than in the control group (Mann–Whitney *U*-test, *p* < 0.001) (Fig. 2).

**Figure 3:**
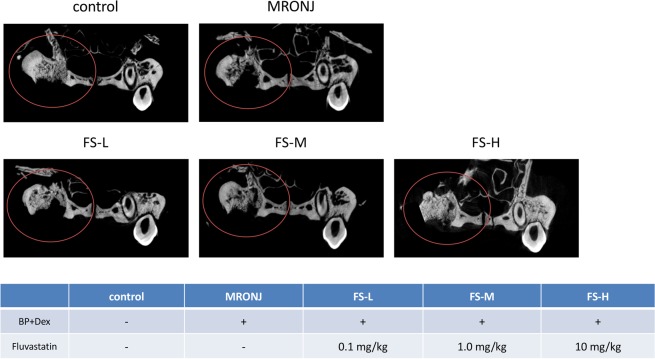
μ-CT findings in the centre of the extraction sockets. There is more new bone formation in the control group than in the MRONJ group. In the FS-H group, new bone formation in the extraction socket is similar to that of the control group.

μ-CT examination showed that the extraction sockets were mostly filled with newly-formed bone in the control group. In the BP + Dex group, the outlines of the extraction sockets remained intact (Fig. 3) and the BV/TV was significantly lower than in the control group (Student’s *t*-test, *p* < 0.001) (Fig. 2).

Thus, it was deemed that the BP + Dex rats constituted an acceptable MRONJ-like model.

### Effects of fluvastatin in MRONJ-like model rats

Next, the ability of fluvastatin to prevent the development of MRONJ-like lesion was investigated. Two weeks after tooth extraction, the sockets of two of the FS-H group rats showed complete restoration of epithelial continuity and formation of underlying connective tissue with no evidence of inflammation or necrotic bone formation. Two sockets showed a small area of bone exposure with inflammatory cell infiltration and bone necrosis. The remaining two sockets showed bone exposure with epithelial discontinuity and necrotic bone. In the FS-M group, complete epithelial continuity had not been restored for any socket, and inflammatory cell infiltration and bone necrosis was detected in all sockets. In the FS-L group, extensive bone exposure with inflammatory cell infiltration and bone necrosis was detected in all sockets (Fig. 1, three rows from right).

New bone formation was detected by μ-CT examination, especially in the FS-H group. In the FS-M group, a small amount of new bone formation was detected in the extraction sockets; however, in the FS-L group the outlines of the extraction sockets were still intact and very little new bone formation was observed (Fig. 3).

**Figure 4:**
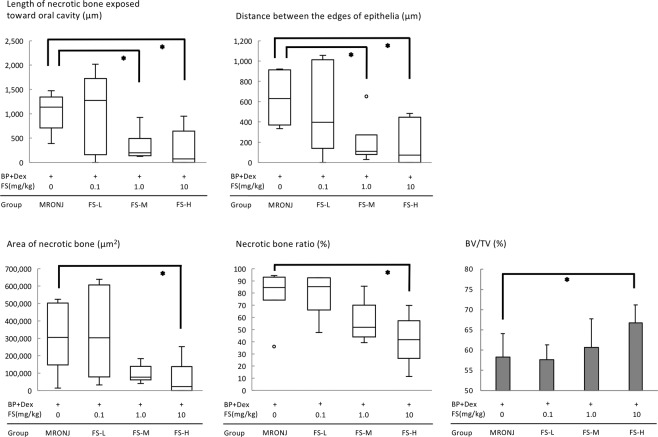
Five variables for evaluating prevention of MRONJ-like lesion by fluvastatin. The length of necrotic bone exposed toward the oral cavity, distance between the edges of the epithelial surfaces, area of necrotic bone, and necrotic bone ratio were measured on histological sections and found to be significantly better in the FS-H group (and in the FS-M group for some variables). Steel’s test. *p < 0.05. BV/TV measured on μ-CT images is significantly larger in the FS-H group than in the MRONJ group. Williams’ test. *p < 0.025.

A significantly shorter length of necrotic bone was exposed toward the oral cavity in the FS-M and FS-H groups than in the MRONJ group (Steel test, MRONJ vs. FS-M: *p* = 0.028; MRONJ vs. FS-H: *p* = 0.041) (Fig. 4). The distance between the edges of the epithelial surfaces was significantly shorter in the FS-M and FS-H groups than in the MRONJ group (Steel test, MRONJ vs. FS-M: *p* = 0.042; MRONJ vs. FS-H: *p* = 0.041) (Fig. 4).

The area of necrotic bone was significantly smaller in the FS-H group than in the MRONJ group (Steel test, MRONJ vs. FS-H: *p* = 0.041) (Fig. 4). The necrotic bone ratio was significantly smaller in the FS-H group than in the MRONJ group (Steel test, MRONJ vs. FS-H: *p* = 0.042) (Fig. 4).

BV/TV calculated on μ-CT images was found to be significantly larger in the FS-H group than in the MRONJ group (Williams’ test, MRONJ vs. FS-H: *p* = 0.021) (Fig. 4).

## Discussion

The null hypothesis of the present study was that fluvastatin has no preventive effect on the development of MRONJ-like lesion. To investigate this hypothesis, we established a MRONJ-like rat model in which we administered a single injection of fluvastatin in the vicinity of tooth extraction sockets to determine whether statins can prevent MRONJ-like lesion.

In the present study, a MRONJ-like rat model was established by administering high dose bisphosphonate and corticosteroid hormone. In the American Association of Oral and Maxillofacial Surgeons, MRONJ is defined as bone exposure that has persisted for longer than eight weeks^8^, but this standard of period cannot be applied for rats and there is no fully elucidated pathophysiology of MRONJ. Therefore, in this study, the validity of this MRONJ-like model was confirmed by the findings that bone exposure, epithelial discontinuity, necrotic bone area, and necrotic bone ratio were significantly larger and there was significantly less new bone formation in the MRONJ group than in the control group. These findings are in accordance with those in a MRONJ-like animal model established by Kaibuchi *et al*.^39^. Of relevance, bisphosphonates are usually used to prevent steroid-induced osteoporosis and one study has found that concurrent use of bisphosphonates with steroids increases the risk of development of MRONJ^43^.

In a previous study, MRONJ-like mice were generated by experimental induction of periapical disease and reportedly developed before the tooth was extracted^44^. In the present study we did not induce any experimental infection to generate MRONJ-like lesion and detected no periodontal or periapical bone necrosis or inflammatory infiltration around the corresponding intact teeth, indicating that the MRONJ-like lesion was triggered by tooth extraction. Using this MRONJ-like rat model, we investigated whether a single local injection of statin reduces the risk of MRONJ-like lesion. The length of necrotic bone exposed toward the oral cavity and the distance between the edges of the epithelial surfaces were significantly shorter in the FS-M and FS-H groups than in the MRONJ group. Statins reportedly promote wound healing both by enhancing epithelial migration^45^ and also by enhancing proliferation of oral epithelial cells and fibroblasts and soft tissue healing at the tooth extraction socket^37^. Since earlier soft tissue closure at the extraction socket in patients receiving bisphosphonates may assist in preventing infection, we expected that these characteristics of statins may have some impacts on the healing of extraction sockets in a MRONJ-like rat model.

The area of necrotic bone and the necrotic bone ratio were significantly smaller in the FS-H group than in the MRONJ group. Statins have been reported to promote angiogenesis^46^, immunomodulation^47^, and antibacterial^34,48,49^ and anti-inflammatory effects^50^. These functions may assist in preventing bone necrosis.

μ-CT examination showed significantly larger hard tissue volumes in the FS-H group, even with induction of MRONJ-like lesion. Since the metabolic effect of statins on bone turnover was reported two decades ago^29^, a number of studies have investigated the mechanisms of this effect. Statins reportedly promote differentiation of osteoblasts^51^ and induction of mRNA expression by bone morphogenetic protein-2^52^. Promotion of bone formation at extraction sockets has also been reported^37^. According to a recent review, the action of statins on bone metabolism may involve many mechanisms, including enhancement of osteoblast proliferation, differentiation, and protection and reduction in osteoclastogenesis^53^.

In the present study, the doses of fluvastatin ranged from 0.1 to 10 mg/kg. In some previous studies, fluvastatin was applied topically to enhance bone metabolism^54,55^. In these studies, the concentrations of fluvastatin ranged from 1.2 to 3.6 mg/kg. In the present study, we used higher concentrations than those; however, in the previous studies fluvastatin was injected for 17 to 25 consecutive days. In contrast, in the present study we only injected fluvastatin once. Histological, histometric and μ-CT findings showed that 10 mg/kg fluvastatin (FS-H group) prevented development of MRONJ-like lesion. Thus, the null hypothesis that “fluvastatin has no preventive effect on the development of MRONJ-like lesion” could be rejected.

Fluvastatin and other statins reportedly have antibacterial effects *in vitro*^33^. An *in vivo* experiment showed that 3% simvastatin-loaded petroleum jelly is an effective treatment for murine methicillin-resistant *Staphylococcus aureus* skin infection^48^. However, given that no *in vivo* studies using fluvastatin as an antibacterial injectable agent have been published, we did not have sufficient data to determine the optimal concentration of fluvastatin as an antibacterial agent in the present study.

Limitations of the present study include that we examined the effects of fluvastatin at only one time point and our study groups were relatively smaller than those in some preceding reports^42,56^. To reduce the number of animals, we employed the minimum number of animals that could maintain statistical soundness. In addition, statins are usually taken orally. Because statins reportedly have low systemic bioavailability due to a first pass effect^57^, we chose to administer the statin by local injection in this study. Indeed, we have shown that a considerable amount of systemically administered statin is required to enhance bone metabolism^58^. Further experiments are mandatory.

In conclusion, our findings suggest that a single local injection of fluvastatin at the time of tooth extraction has the potential to reduce the chance of developing MRONJ-like lesion in a rat model.

## Data Availability

The primary data that support the results described here are available from the corresponding author upon reasonable request.
